# Comments on: Risk of dyslipidemia in chronic hepatitis B patients taking tenofovir alafenamide: a systematic review and meta-analysis

**DOI:** 10.1007/s12072-024-10642-0

**Published:** 2024-03-02

**Authors:** Yan Wang, Xiu Sun, Junyan Zhang, Qi Mei

**Affiliations:** 1 Department of Infection, Shanxi Bethune Hospital, Shanxi Academy of Medical Sciences, Tongji Shanxi Hospital, Third Hospital of Shanxi Medical University, Taiyuan, China; 2 Department of Clinical Epidemiology and Evidence-Based Medicine, Shanxi Bethune Hospital, Shanxi Academy of Medical Sciences, Tongji Shanxi Hospital, Third Hospital of Shanxi Medical University, Taiyuan, China; 3 Cancer Center, Shanxi Bethune Hospital, Shanxi Academy of Medical Sciences, Tongji Shanxi Hospital, Third Hospital of Shanxi Medical University, Taiyuan, China

Dear Editor

With sincere interest, we have carefully reviewed the article “Risk of dyslipidemia in chronic hepatitis B patients taking tenofovir alafenamide: a systematic review and meta-analysis,” published in your esteemed journal by Hwang et al. [1]. This meta-analysis revealed that individuals receiving the tenofovir alafenamide(TAF) regimen demonstrated notably higher elevations in various lipid parameters.

We express our gratitude to the authors for their dedication to this paper. While acknowledging the merit, we seek further clarifications, as addressing these concerns may contribute to a more comprehensive understanding of the presented findings.

Our first concern pertains to the heterogeneity. As outlined in the meta-analysis, the enrolled studies comprised two randomized controlled trials (RCTs), three prospective cohorts, and seven retrospective cohorts. Notably, the *I*^2^ statistic exceeded 45% for all the results, and the majority (26 out of 28) were with 80% and above *I*^2^ statistics (refer to Table 2–4) [1]. Previous studies proposed that an *I*^2^ surpassing 75% indicated high heterogeneity [2], while 50% suggested a significant heterogeneity [3]. The Cochrane Handbook clarified, “Clinical variation will lead to heterogeneity”.^1^ As presented in the appendix of Hwang et al. study,^2^ the authors put RCTs together with cohort studies to calculate the effect size, while we thought different types of studies were one of the critical factors contributing to heterogeneity. Even meta-regression should also analyze studies of the same category.

Our second concern revolves around potential biases. As elucidated by the Lancet [4], a fundamental limitation of cohort studies was the selection bias. Additionally, analyses of cohort data should address and mitigate potential confounding factors [4]. We noted that those enrolled observational studies did not incorporate multivariate adjustments when examining the changes in lipid levels. This omission increases the likelihood that this meta-analysis's final results may be subject to bias. Notably, one enrolled study [5] reported changes in total cholesterol levels in Supplementary Table 8 from their Appendix,^3^ not in triglyceride levels, as reported in Table 2 of the meta-analysis [1].

Our final concern pertains to the evaluation tool used for assessing the risk of bias, as presented in the appendix of Hwang et al. study^2^. According to the Cochrane Handbook,^4^ the Cochrane risk-of-bias tool is designed for assessing specific outcomes in randomized trials. Therefore, its application to observational studies is not aligned with its intended use. Moreover, we find it inappropriate that the authors asserted that all observational studies had low risks of bias concerning blinding outcome data^2^. As observational studies were not designed with blinding protocols, the bias in this aspect should at least be categorized as unclear.^5^ We recommend that the authors reconsider the choice of evaluation tools and accurately reflect the inherent limitations of blinding in observational studies.

We appreciate discussing this impactful research and hope our considerations rigorously advance our understanding of TAF’s dyslipidemia risks. Please accept our heartfelt gratitude to the authors and the editors for enabling valuable scientific exchange essential for the collective advancement of clinical knowledge.

## Footnotes

1 https://training.cochrane.org/handbook/current/chapter-10#section-10-10.

2 https://link.springer.com/article/10.1007/s12072-023-10528-7#availability-of-data-and-materials.

3 https://onlinelibrary.wiley.com/action/downloadSupplement?doi=10.1111%2Fhepr.13726&file=hepr13726-sup-0001-suppl-data.pdf.

4 https://training.cochrane.org/handbook/archive/v6.3/chapter-08.

5 https://training.cochrane.org/handbook/current.

## References

[1] Hwang EG, Jung EA, Yoo JJ, Kim SG, Kim YS (2023). Risk of dyslipidemia in chronic hepatitis B patients taking tenofovir alafenamide: a systematic review and meta-analysis. Hepatol Int.

[2] Borenstein M. In a meta-analysis, the I-squared statistic does not tell us how much the effect size varies. J Clin Epidemiol (United States). 2022;152:281–4. 10.1016/j.jclinepi.2022.10.003 36223816

[3] Higgins JP, Thompson SG, Deeks JJ (2003). Measuring inconsistency in meta-analyses. BMJ (England)..

[4] Grimes DA, Schulz KF (2002). Cohort studies: marching towards outcomes. Lancet (England)..

[5] Hosaka T, Suzuki F, Kobayashi M, et al. Renal safety and biochemical changes for 2 years after switching to tenofovir alafenamide from long-term other nucleotide analog treatment in patients with chronic hepatitis B. Hepatol Res (Netherlands). 2022;52(2):153–64. 10.1111/hepr.13726 34687121

